# Umbilical cord blood concentration of connecting peptide (C-peptide) and pregnancy outcomes

**DOI:** 10.1186/s12884-022-05081-4

**Published:** 2022-10-12

**Authors:** Atrin Niknam, Fahimeh Ramezani Tehrani, Samira Behboudi-Gandevani, Maryam Rahmati, Mehdi Hedayati, Mehrandokht Abedini, Faegheh Firouzi, Farahnaz Torkestani, Mehdi Zokaee, Fereidoun Azizi

**Affiliations:** 1 Reproductive Endocrinology Research Center, Research Institute for Endocrine Sciences, Shahid Beheshti University of Medical Sciences, Tehran, Iran; 2 Faculty of Nursing and Health Sciences, Nord University, Bodø, Norway; 3 Cellular and Molecular Research Center, Research Institute for Endocrine Sciences, Shahid Beheshti University of Medical Sciences, Tehran, Iran; 4 Infertility and cell therapy office, Transplant & Disease Treatment Center, Ministry of Health and Medical Education, Tehran, Iran; 5 Tehran Medical Branch, Islamic Azad University, Tehran, Iran; 6 Shahed University of Medical Sciences, Tehran, Iran; 7 Senior Executive of Public Health, Kurdistan University of Medical Sciences, Sanandaj, Iran; 8 Endocrine Research Center, Research Institute for Endocrine Sciences, Shahid Beheshti University of Medical Sciences, Tehran, Iran

**Keywords:** C-peptide, Pregnancy outcomes, Macrosomia, Gestational diabetes mellitus

## Abstract

**Background:**

C-peptide offers potential as a marker to indicate childhood metabolic outcomes. Measuring C-peptide concentration might have better future utility in the risk stratification of neonates born to overweight or diabetic mothers. Prior research has tried to bring this matter into the light; however, the clinical significance of these associations is still far from reach. Here we sought to investigate the associations between fetomaternal metabolic variables and umbilical cord blood C-peptide concentration.

**Methods:**

For the present study, 858 pregnant women were randomly selected from among a sub-group of 35,430 Iranian pregnant women who participated in a randomized community non-inferiority trial of gestational diabetes mellitus (GDM) screening. Their umbilical cord (UC) blood C-peptide concentrations were measured, and the pregnancy variables of macrosomia/large for gestational age (LGA) and primary cesarean section (CS) delivery were assessed. The variation of C-peptide concentrations among GDM and macrosomia status was plotted. Due to the skewed distribution of C-peptide concentration in the sample, median regression analysis was used to identify potential factors related to UC C-peptide concentration.

**Results:**

In the univariate model, positive GDM status was associated with a 0.3 (95% CI: 0.06 − 0.54, p = 0.01) increase in the median coefficient of UC blood C-peptide concentration. Moreover, one unit (kg) increase in the birth weight was associated with a 0.25 (95% CI: 0.03 − 0.47, p = 0.03) increase in the median coefficient of UC blood C-peptide concentration. In the multivariate model, after adjusting for maternal age, maternal BMI, and macrosomia status, the positive status of GDM and macrosomia were significantly associated with an increase in the median coefficient of UC blood C-peptide concentration (Coef.= 0.27, 95% CI: 0.13 − 0.42, p < 0.001; and Coef.= 0.34, 95% CI: 0.06 − 0.63, p = 0.02, respectively).

**Conclusion:**

UC blood concentration of C-peptide is significantly associated with the incidence of maternal GDM and neonatal macrosomia. Using stratification for maternal BMI and gestational weight gain (GWG) and investigating molecular markers like Leptin and IGF-1 in the future might lay the ground to better understand the link between metabolic disturbances of pregnancy and UC blood C-peptide concentration.

**Supplementary Information:**

The online version contains supplementary material available at 10.1186/s12884-022-05081-4.

## Introduction

Human connecting peptide (C-peptide) is part of the insulin precursor molecule. It is necessary for the correct folding of insulin amino acid chains to their final form and is secreted in equimolar concentrations with insulin [1]. Any process affecting insulin secretion could also affect C-peptide concentration; as a result, it might be considered a proxy variable reflecting metabolic alterations [2]. Due to the detrimental fetomaternal impact of metabolic aberrations on the gravida and her child, growing attention to the correlation between these aberrations and cord blood C-peptide concentration has emerged in recent years [3–7].

Maternal metabolic aberrations increase the likelihood of gestational disturbances, adverse neonatal outcomes, childhood disorders of glucose metabolism, and childhood obesity [3, 8–12]. The effect of metabolic aberrations on the fetus could lead to long-term consequences, including diabetes, hypertension, obesity, cardiovascular dysfunction, and metabolic syndrome [3, 8, 12, 13]. Simultaneously, these aberrations might be correlated with the concentration of maternal and cord blood circulating analytes. This has rendered cord blood C-peptide concentration a potential complementing marker reflecting the status of fetomaternal metabolism [14–16]. For instance, maternal obesity and excessive gestational weight gain (GWG) contribute to increased maternal insulin secretion, elevated risk of developing gestational diabetes mellitus (GDM), and possibly increased cord blood insulin and C-peptide concentration [7, 17, 18]. Moreover, increased maternal plasma glucose might trigger fetal pancreas hypertrophy -a prerequisite for fetal macrosomia and hyperinsulinemia- and might increase cord blood insulin and C-peptide concentration [19, 20].

C-peptide concentration does offer potential as a marker to indicate childhood metabolic outcomes. Moreover, C-peptide concentration measurement might improve risk stratification in neonates born to overweight or diabetic mothers, allowing appropriate resource allocation to those most at risk. Prior research has tried to bring this matter into the light, as it has investigated the association between C-peptide concentration and maternal BMI, diabetes [12], GWG [18], and fetal overgrowth [21]. However, the clinical significance of these associations is still far from reach. Moreover, a few population-based studies were conducted to assess the practicality of C-peptide concentration measurement in pregnant women [3–6]. Using a subset of a population-based cohort in Iran, called the Persian Gulf Study, we sought to investigate the associations between fetomaternal metabolic variables and umbilical cord blood C-peptide concentration.

## Materials and methods

### Study participants

The present study participants were selected from Iranian pregnant women who participated in a randomized community-based non-inferiority trial of gestational diabetes mellitus (GDM) screening. The details of this study were published before [22]. Briefly, a total of 35,430 pregnant women in their first pregnancy trimester were recruited from five different geographic regions of Iran. The pregnant women who were less than 18 years old, had preexisting diabetes or other chronic disorders, were uncertain about the date of their last menstrual period (LMP), and did not have an ultrasound examination during their 6th to 14th weeks of gestation were all excluded. Along with standard prenatal care, all participants were scheduled to have two phases of GDM screening in the first and second pregnancy trimesters. GDM status was screened based on fasting plasma glucose (FPS) in the first and either a one-step or a two-step screening method second trimester of pregnancy. All participants were followed until delivery, and their outcomes were recorded in detail. Those with a GDM diagnosis received specific care according to the recommended guidelines for GDM treatment [23]. The main findings of this trial were published before [24].

A sub-group of the abovementioned trial participants was selected for the present study, and their umbilical cord (UC) blood C-peptide concentrations were assessed. In this regard, we randomly selected 858 pregnant women assigned to protocol A. Protocol A is consistent with the IADPSG recommendations for GDM screening. This protocol defines first trimester GDM as 5.1 mmol/L < FPG < 7 mmol/L. All participants with first-trimester FPG < 5.1 mmol/L were re-screened during the 24th -28th weeks of gestation using a single step 2-h 75 g oral glucose tolerance test (OGTT). GDM was diagnosed when FPG values reached or exceeded any of the mentioned values (FPG ≥ 5.1 mmol/L; 1 h-pp BG ≥ 10 mmol/L; 2 h-pp BG ≥ 8.5 mmol/L). Women with GDM diagnosed in the first or second pregnancy trimester received appropriate treatment. The treatment was initiated with lifestyle modifications (medical nutrition therapy and physical activity adjustment) and blood glucose monitoring to achieve the target values of FPG = 95 mg/dL, 1 h-pp BG = 140 mg/dL or 2 h-pp BG = 120 mg/dL. If they did not achieve the glycemic goals within two weeks, pharmacologic therapy was offered by expert physicians (obstetricians, internists, or endocrinologists) as the second level of the healthcare delivery system.

### Fetomaternal variables

Macrosomia/large for gestational age (LGA), primary cesarean section (CS) delivery, preterm birth before 37 weeks of gestation, admission to the neonatal intensive care unit (NICU), neonatal hypoglycemia, neonatal hypocalcemia, neonatal hyperbilirubinemia, preeclampsia, birth trauma, low birth weight (LBW), and intrauterine fetal demise (IUFD) are the variables that were investigated in this study.

### Definitions

Macrosomia/large for gestational age (LGA) was defined as birth weight > 4000 g or fetal weight > 90th percentile for a given gestational age using ultrasound biometry for estimating the fetal weight and multinational World Health Organization (WHO) fetal growth chart for defining the percentile [25]. Primary cesarean section was defined as the cesarean deliveries out of all births to women who had not had a previous cesarean delivery. Hypoglycemia was defined as plasma glucose concentration < 2.6 mmol/l in the first 48 h after delivery [26]; hyperbilirubinemia was determined by a value greater than the 95th percentile for any given point after birth [27]; Preeclampsia was defined as high blood pressure after 20 weeks of gestation, meaning a systolic blood pressure (SBP) ≥ 140 mmHg or a diastolic blood pressure (DBP) ≥ 90 mmHg on two separate occasions at least four hours apart in women with previously normal blood pressure, and proteinuria ≥ 300 mg per 24 h urine collection, or protein/creatinine ratio ≥ 0.3, or dipstick reading of 1 + or more if other quantitative methods were not available [28]; Preterm birth was defined as when delivery occurs between 20 and 37 weeks of gestation [29]; Birth trauma was defined as brachial plexus palsy or clavicular, humeral, or skull fracture; Low birth weight (LBW) was defined as a weight < 2500 g at birth regardless of gestational age.

### Measurements

The umbilical blood was drawn immediately after delivery. To minimize glycolysis within erythrocytes and falsely lower glucose values, sodium fluoride-containing tubes were used to collect the samples, were kept on ice until plasma separation within one hour, and were frozen at -20 °C. Samples arrived deep-frozen at the Central Laboratory of the Research Institute for Endocrine Sciences. The amount of dry ice required was ascertained for each field center. The Central Laboratory was notified of shipping details. On arrival at the Central Laboratory, samples were assessed to ensure they were frozen. The samples were placed in freezer boxes for storage at -80 °C. All samples with any degree of hemolysis were excluded for C-peptide measurement (n = 16). C-peptide was measured using an Enzyme-Linked Immunosorbent Assay (ELISA C-peptide Kit, DRG Diagnostics GmbH). This kit uses a solid-phase competitive assay. The capture antibody was coated on a well, and the sample’s C-peptide competes with HRP-conjugated C-peptide lyophilized standards after preparation according to the kit’s inset were used to obtain the standard curve. After incubation, we measured the optical density with an ELISA reader and calculated the C-peptide concentrations according to the standard curve. The assay sensitivity was 0.064 ng/ml, and samples greater than 16 ng/mL were re-assayed at 1:10 dilution.

### Statistical analysis

Continuous variables were checked for normality using the Shapiro–Wilk test; those with normal distribution were expressed as mean (standard deviation), and non-normal distributed variables were expressed as median (interquartile range). Categorical variables were expressed as percentages. The variation of C-peptide concentrations according to the GDM and macrosomia status was plotted. Because the C-peptide concentrations were not normally distributed, the median regression model was used to assess the effect of some variables on the C-peptide. Median regression is a flexible and robust methodology in which coefficients reveal the effect of a unit change in the covariate on the median of the response distribution [30]. Univariate median regression analysis with a 95% confidence level was used to identify potential risk factors related to C-peptide. Variables with a P-value of less than 0.2 in the univariate analysis were then added to the final multivariate model. Statistical analysis was performed using the software package STATA (version 13; STATA Inc., College Station, TX, USA); the significance level was set at P < 0.05, and the confidence interval was at 95%.

## Results

Baseline characteristics of the study participants are presented in Table 1.

**Table 1:** Baseline characteristics of the study participants

variables		N = 842
Age (years) ^a^		30.23 (5.85)
Maternal weight at first (kg) ^a^		70.10 (11.81)
Maternal BMI (kg/m^2^) ^a^		27.41(4.75)
Gravidity ^b^		2 (1–3)
Parity ^b^		1 (0–2)
	> 1^c^	433 (58.0)
GWG (kg) ^b^		10.9 (7.77–13.4)
UC C-peptide (ng/ml) ^b^		1.58 (0.93–2.24)
Gestational age at delivery (weeks) ^a^		38.93 (2.96)
Type of delivery (CS)^c^		17 (2.29)
GDM ^c^		99 (11.84)
Preeclampsia ^c^		29 (3.87)
End of pregnancy ^c^		
	Term	715 (95.46)
	Abortion	6 (0.80)
	Preterm	28 (3.74)
Infant sex (male)^c^		108 (49.54)
Birth weight (gr) ^a^		3343.8 (421.12)
Macrosomia ^c^		50 (6.0)
LBW ^c^		11 (5.02)
NICU admission ^c^		14 (1.67)
Fetal hypoglycemia ^c^		2 (0.24)
Fetal hypocalcemia ^c^		3 (0.36)
Birth trauma ^c^		2 (0.24)
IUFD ^c^		3 (0.36)

^a^ Mean (standard deviation); ^b^ Median (inter-quartile range); ^c^ Number (Percentage).

Abbreviations: BMI, Body mass index; GWG, Gestational weight gain; UC, Umbilical cord; CS, Cesarean section; GDM, Gestational diabetes mellitus; NICU, Neonatal intensive care unit; IUFD, Intrauterine fetal demise; LBW, Low birth weight.

**Fig. 1:**
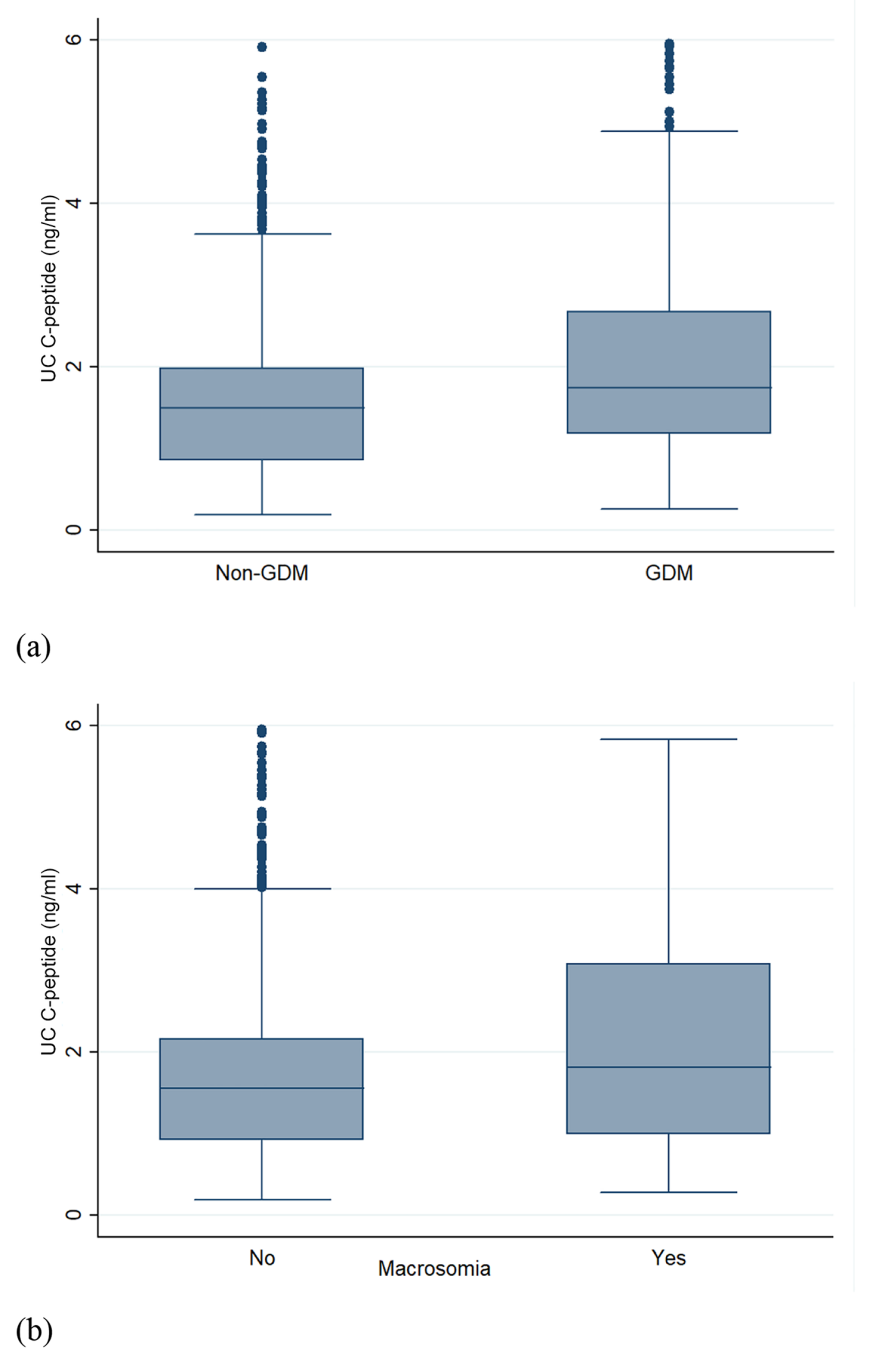
Box plots for comparison of UC C-peptide concentration (ng/ml) among the study participants with respect to their status of GDM and macrosomia. (a) C-peptide concentration among GDM vs. non-GDM women. (b) C-peptide concentration among women who delivered infants with macrosomia vs. women who delivered normal weight infants. (Abbreviations: UC, Umbilical cord; GDM, Gestational diabetes mellitus)

The comparisons of the associations between UC (umbilical cord) blood C-peptide concentrations and different pregnancy variables are presented in Fig. 1. Women with GDM had a higher median (Q25 − Q75) UC blood C-peptide concentration compared to non-GDM women (1.74 ng/ml (1.18 − 2.67) and 1.49 ng/ml (0.85 − 1.98), respectively; p < 0.001). Moreover, women delivering a macrosomic baby had a slightly significantly higher median (Q25 − Q75) UC blood C-peptide concentration compared to those with normal weight babies (1.81 ng/ml (0.99 − 3.08) and 1.56 ng/ml (0.92 − 2.16), respectively; p = 0.06).

Univariate and multivariate median regression models for the C-peptide outcome are presented in Table 2. In the univariate model, positive GDM status was associated with a 0.3 (95% CI: 0.06 − 0.54, p = 0.01) increase in the median coefficient of UC blood C-peptide concentration. Moreover, one unit (kg) increase in the birth weight was associated with a 0.25 (95% CI: 0.03 − 0.47, p = 0.03) increase in the median coefficient of UC blood C-peptide concentration. Multiparity was negatively associated with the median coefficient of UC blood C-peptide concentration (-0.16, 95% CI: -0.30, -0.02, p = 0.03). Our analyses indicated no significant association between median C-peptide concentration in the cord blood and maternal age, maternal BMI, gestational age at delivery, gestational weight gain (GWG), infant sex, and macrosomia status as a binary variable (Table 2). In the multivariate model, after adjusting for maternal age, maternal BMI, and macrosomia status, only the association between GDM status and median UC blood C-peptide concentration remained significant (Coef.= 0.27, 95% CI: 0.13 − 0.42, p < 0.001). This model also indicated a significant association between the positive status of macrosomia and an increase in the median coefficient of UC blood C-peptide concentration (Coef.= 0.34, 95% CI: 0.06 − 0.63, p = 0.02). This association was not observed in the univariate model.

**Table 2:** Median regression model for UC blood concentration of C-peptide

Variable	Coef. (95%CI)	p-value
Univariate model		
GDM	0.30 (0.06,0.54)	**0.01**
Birth weight (kg)	0.25 (0.03,0.47)	**0.03**
Parity ≥1	-0.16 (-0.30, -0.02)	**0.03**
Age (y)	-0.004 (-0.02,0.01)	0.5
BMI (kg/m^2^)	-0.01 (-0.03, 0.004)	0.12
GWG (kg)	0.04 (-0.06,0.14)	0.4
Gestational age (weeks)	0.02 (-0.12,0.16)	0.7
Macrosomia	0.26(-0.05,0.57)	0.1
Sex (male)	0.06(-0.14,0.26)	0.56
Multivariate model*		
GDM	0.28 (0.10,0.46)	**0.002**
Macrosomia	0.39 (0.08,0.69)	**0.01**
Parity ≥1	-0.13 (-0.32,0.05)	0.1
Age (y)	-0.01 (-0.02,0.01)	0.7
BMI (kg/m^2^)	-0.01 (-0.03,01)	0.4

*In spite of the p-value < 0.2, birth weight was not included in the multivariate model, because of its strong association with macrosomia. Moreover, macrosomia as a binary variable was a better fit for the model. Maternal age and BMI were also added to the multivariable model due to their importance in the literature.

Abbreviations: UC, Umbilical cord; BMI, Body mass index; GWG, Gestational weight gain; GDM, Gestational diabetes mellitus.

## Discussion

The present study indicates that umbilical cord (UC) blood C-peptide concentration is significantly associated with the GDM status of the mother and the macrosomic status of the neonate.

Experimental studies indicated a link between C-peptide and decreased formation of radical oxygen species (ROS) [31], increased inhibition of apoptosis, decreased expression of adhesion molecules, and the inflammatory pathway [32]. However, as the C-peptide receptor and its mechanism of binding are yet to be determined, studies are inconclusive regarding the actual biologic effects of this peptide [33]. Being connected to insulin precursor molecule, thus, being secreted in equimolar concentrations as insulin [2] might be the crucial rationale to consider C-peptide as a potential marker reflecting metabolic outcomes; moreover, C-peptide has a longer half-life (5 to 6-fold longer) compared to insulin [1], which makes C-peptide measurement a better candidate. As a result, its measurement in metabolic disturbances such as metabolically unhealthy pregnancies might be rewarding.

Several studies have investigated the associations between C-peptide and unfavorable pregnancy outcomes [4–7, 12, 34–37]. It is proposed that neonatal predisposition to insulin resistance and hypoglycemia might initially present as an increased concentration of C-peptide in the umbilical cord (UC) blood [34, 38]. Fetal insulin resistance, fetal growth, and therefore, the risk of macrosomia might be positively associated with insulin and C-peptide concentration in UC blood [6, 7, 35]. Moreover, neonatal adiposity is strongly associated with C-peptide concentration in the maternal blood [7, 39]. C-peptide promotes the growth of peri-peritoneal adipose tissue. The disproportionate growth in the adipose tissue increases the risk of childhood disorders of glucose metabolism [40]. A recent population-based cohort assessed the maternal C-peptide concentration of almost 6000 pregnant women in Brazil; there was no significant association between maternal C-peptide and neonatal birth weight [5]. This finding suggests that it might be incorrect to equivalently interpret C-peptide concentrations in the maternal, UC, and neonatal blood. Therefore, various reported associations between C-peptide and fetomaternal outcomes might be due to C-peptide multiple sources of measurement.

Several mechanisms might be in play in the correlation between C-peptide concentrations in the maternal, UC, and neonatal blood. Gestational weight gain (GWG) might affect UC C-peptide concentration [17]. It is also proposed that GWG and UC C-peptide are associated with neonatal birth weight [21, 41]. Moreover, neonatal birth weight might be affected by maternal insulin sensitivity [40]. As a result, maternal insulin sensitivity was suggested as a potential mediator of the association between GWG and UC C-peptide [17]. In other words, maternal insulin sensitivity might affect how GWG variations translate into UC C-peptide concentration variations. Thus, maternal insulin sensitivity might partly explain the lack of significance in our study’s association between UC C-peptide and GWG. This might be one mechanism that determines how maternal, UC, and neonatal C-peptide concentrations are associated. Another possible mechanism might arise from the different placenta conditions between primiparas and multiparas. The insulin-like growth factor-1 (IGF-1) concentration is lower in primiparas [42, 43]. IGF-1 is a hormone associated with UC C-peptide, neonatal birth weight, and adiposity indices [20, 43]. IGF-1 being associated with parity and with UC C-peptide might partly explain the significant association we observed between UC C-peptide and parity. Moreover, one study has found that the association between UC C-peptide and neonatal birth weight will lose significance after adjusting for IGF-1 [7]. Therefore, similar to maternal insulin sensitivity, IGF-1 might affect UC C-peptide to the extent that the association between C-peptide concentrations in the maternal, UC, and neonatal blood changes in direction or magnitude. Lastly, it is noteworthy that the possibility of these mediators affecting the associations between C-peptide and other fetomaternal variables cannot be ruled out. The lack of measurement of IGF-1 in the present study makes us unable to investigate this hypothesis.

A significant association was reported between abnormal oral glucose tolerance tests (OGTT) during pregnancy with an abnormal C-peptide concentration [3]. Mothers with GDM have a lower umbilical cord (UC) concentration of glucose and a simultaneous higher UC concentration of insulin and C-peptide [12, 40]. We observed that GDM status was positively associated with UC blood concentration of C-peptide. Moreover, a case-control study on 83 newborns reported that among the infants born to non-GDM mothers, UC blood C-peptide concentration was comparable between large-for-gestational-age (LGA) and appropriate-for-gestational-age (AGA) [7]. It is noteworthy that maternal or neonatal C-peptide concentrations might have differed between LGA and AGA infants. Apart from that, we observed that UC C-peptide was associated with macrosomia, but the interaction between GDM and macrosomia did not reach statistical significance in our study. It seems that the association between UC C-peptide and macrosomia might not be highly influenced by GDM status; however, maternal or fetal adiposity status might affect this association. Maternal obesity, per se, might result in higher insulin and C-peptide secretion in the maternal and UC blood and is associated with neonatal birth weight [6, 7, 39].

The literature indicates strong associations between maternal BMI, UC C-peptide, and neonatal birth weight [6, 7, 39]. These associations might be regulated by an adipocytokine called Leptin. Leptin is involved in satiety and energy regulation through appetite control [44], which might be associated with C-peptide concentration [36]. Besides Leptin’s role in energy regulation, its resemblance to IL-6 brought up the idea of its role as an inflammatory cytokine [45]. Moreover, Leptin modulates the innate immune response and stimulates the releases of proinflammatory cytokines and prostaglandins from maternal adipose tissue and the placenta, specifically TNF-α, IL-6, IL-1 β, and PGE [46]. Higher concentrations of Leptin, insulin, and C-peptide are strongly associated with excessive GWG and obesity [36]. This brings up the assumption that Leptin might affect the association between UC C-peptide concentration and GWG, as well as UC C-peptide and macrosomia.

In the present study, UC C-peptide concentration was not affected by infant sex. One study on 582 Irish women and their children reported higher concentrations of C-peptide and Leptin in the UC blood of the female infants and lower insulin resistance in their mothers [47]. The authors hypothesized that due to their smaller placental mass, female fetuses might possess lower concentrations of placental hormones associated with increased insulin resistance, such as placental lactogen [48]. Of note, the mentioned study was nested on a cohort that enrolled women with a history of giving birth to a macrosomic infant, meaning they were not primiparas. As stated, multiparas might have higher concentrations of IGF-1, which means higher concentrations of UC C-peptide. It is possible that there actually is a difference in the concentrations of UC C-peptide between different sexes. However, it only becomes significant when a higher concentration of UC C-peptide is present. Moreover, IGF-1 is positively associated with female sex and UC C-peptide [20, 49]; therefore, IGF-1 might influence the association between infant sex and UC C-peptide. Due to the difference in the participant’s parity between the two studies, the mean concentrations of IGF-1 might differ. Considering the role of IGF-1, its different concentrations might justify the various associations of infant sex and UC C-peptide concentrations between the studies.

Strengths of the present study include the population-based nature of the investigation, conducting all C-peptide measurements in a single center to decrease the intra-assay variation, and the adjustments made for the main confounders. However, this study also faced some limitations. First, we did not measure specific adiposity markers, including skin-fold thickness (SFT), total fat mass, fat-free mass, and length gain, as these markers determine neonatal body composition more accurately [50]. Second, had we measured markers like Leptin and IGF-1, we might have been able to unravel the mediator in the association between GWG and UC blood C-peptide concentration and the reason behind the lack of association between these two factors in our study in contrast to the previous evidence. Third, our sample was not adequate to stratify our participants based on their BMI and GWG; this approach might have led us to better understand the role of maternal BMI and GWG in the correlation between UC C-peptide concentration and fetomaternal outcomes.

## Conclusion

In a population-based cohort study performed in different geographical regions of Iran, we demonstrate that UC blood concentration of C-peptide is significantly associated with the incidence of maternal GDM and neonatal macrosomia. Measuring C-peptide concentration has the potential to improve the risk stratification of neonates born to overweight or diabetic mothers. Though it seems promising, the existing evidence supporting the link between UC blood C-peptide concentration and fetomaternal metabolic outcomes is not yet adequate. For future studies, using stratification for maternal BMI and GWG, measuring variables in different trimesters, measuring maternal, UC, and neonatal C-peptide concentrations to compare their correlations with fetomaternal variables, and investigating molecular markers like Leptin and IGF-1 might lay the ground to understand better the link between UC C-peptide concentration and fetomaternal metabolic outcomes.

## Electronic supplementary material

Below is the link to the electronic supplementary material.

Supplementary Material 1

## Data Availability

The datasets used and/or analyzed during the current study are available from the corresponding author on reasonable request (ramezani@endocrine.ac.ir).

## Abbreviations

C-peptide: Connecting peptide
GDM: Gestational diabetes mellitus
UC: Umbilical cord
LGA: Large-for-gestational-age
CS: Cesarean section
BMI: Body mass index
GWG: Gestational weight gain
IGF-1: Insulin-like growth factor-1
LMP: Last menstrual period
FPG: Fasting plasma glucose
OGTT: Oral glucose tolerance test
BG: Blood glucose
NICU: Neonatal intensive care unit
LBW: Low-birth-weight
IUFD: Intrauterine fetal demise
WHO: World health organization
SBP: Systolic blood pressure
DBP: Diastolic blood pressure
ROS: Reactive oxygen species
AGA: Appropriate-for-gestational-age
IL-6: Interleukin-6
TNF-α: Tumor necrosis factor-α
IL-1: Interleukin-1
PGE: Prostaglandin E
SFT: Skin fold thickness
IADPSG: The International Association of Diabetes and Pregnancy Study Groups
