# Supplementary material for: Umbilical cord blood concentration of connecting peptide (C-peptide) and pregnancy outcomes
Source: BMC Pregnancy Childbirth. 2022 Oct 12;22:764. doi: 10.1186/s12884-022-05081-4 (PMC9559016; doi:10.1186/s12884-022-05081-4)
Supplement: Supplementary file 1 — Supplementary Material 1 [file 12884_2022_5081_MOESM1_ESM.docx]

**[Supplementary] Table 1**. Baseline characteristics of the study participants.

| **variables** | | **Total (n= 842)** | **GDM (n=99)** | **Non- GDM(n=743)** | **p-value^¥^** |
| --- | --- | --- | --- | --- | --- |
| Age (years) ^a^ | | 30.23 (5.85) | 31.92(5.9) | 29.9(5.8) | 0.002 |
| Maternal weight at first (kg) ^a^ | | 70.10 (11.81) | 72.9(8.6) | 69.5(12.3) | 0.5 |
| Maternal BMI (kg/m^2^) ^a^ | | 27.41(4.75) | 29.8(3.9) | 27.0(4.8) | 0.2 |
| Gravidity ^b^ | | 2.0 (1-3) | 2.0 (1-3) | 2.0 (1-3) | 0.1 |
| Parity ^b^ | | 1 (0-2) | 2(2-3) | 1(0-1) | 0.005 |
|  | >1^c^ | 433 (58.0) | 62(63.3) | 371(57.2) | 0.3 |
| GWG (kg) ^b^ | | 10.9 (7.77-13.4) | 10.2(6.1-10.9) | 11.3(8.3-13.4) | 0.2 |
| UC C-peptide (ng/ml) ^b^ | | 1.58 (0.93-2.24) | 1.85 (1.50-2.86) | 1.55(0.86-2.12) | <0.001 |
| Gestational age at delivery (weeks) ^a^ | | 38.93 (2.96) | 38.39(4.35) | 39.01(2.28) | 0.05 |
| Type of delivery (CS)^c^ | | 17 (2.29) | 5(5.1) | 12(1.9) | 0.04 |
| GDM ^c^ | | 99 (11.84) | - | - | - |
| Preeclampsia ^c^ | | 29 (3.87) | 6(6.1) | 23(3.5) | 0.2 |
| End of pregnancy ^c^ | |  |  |  | 0.2 |
|  | Term | 715 (95.46) | 92(92.9) | 623(95.8) |  |
|  | Abortion | 6 (0.80) | 2(2.0) | 4(0.6) |  |
|  | Preterm | 28 (3.74) | 5(5.0) | 23(3.5) |  |
| Infant sex (male)^c^ | | 108 (49.54) | 10(41.7) | 98(50.5) | 0.4 |
| Birth weight (gr) ^a^ | | 3343.8 (421.12) | 3302.3(209.3) | 3348.9(440.2) | 0.6 |
| Macrosomia ^c^ | | 50 (6.0) | 6(6.2) | 44(6.0) | 0.9 |
| LBW ^c^ | | 11 (5.02) | 0 | 11(5.6) | 0.2 |
| NICU admission ^c^ | | 14 (1.67) | 3(3.0) | 11(1.5) | 0.3 |
| Fetal hypoglycemia ^c^ | | 2 (0.24) | 1(1.0) | 1(0.1) | 0.09 |
| Fetal hypocalcemia ^c^ | | 3 (0.36) | 0 | 3(0.4) | 0.5 |
| Birth trauma ^c^ | | 2 (0.24) | 0 | 2(0.3) | 0.6 |
| IUFD ^c^ | | 3 (0.36) | 0 | 3(0.4) | 0.5 |
| **^¥^** Comparison between GDM and non-GDM groups. Independent t-test or Pearson’s Chi-squared test for continuous and categorical data was applied, respectively. The Mann–Whitney U test was applied to compare variables with skewed distribution.  ^a^ Mean (standard deviation); ^b^ Median (inter-quartile range); ^c^ Number (Percentage).  Abbreviations: BMI, Body mass index; GWG, Gestational weight gain; UC, Umbilical cord; CS, Cesarean section; GDM, Gestational diabetes mellitus; NICU, Neonatal intensive care unit; IUFD, Intrauterine fetal demise; LBW, Low birth weight. | | | | | |
